# A Domain Generalization Method for EEG Based on Domain-Invariant Feature and Data Augmentation

**DOI:** 10.34133/cbsystems.0508

**Published:** 2026-02-24

**Authors:** Jing Jin, Junxian Li, Xiaochuan Pan, Ren Xu, Andrzej Cichocki, Wenli Du, Feng Qian

**Affiliations:** ^1^ Key Laboratory of Smart Manufacturing in Energy Chemical Process, Ministry of Education, East China University of Science and Technology, Shanghai 200237, China.; ^2^ School of Mathematics, East China University of Science and Technology, Shanghai 200237, China.; ^3^ The Department of Engineering, University of Applied Sciences Campus Vienna, 1100 Vienna, Austria.; ^4^ The Systems Research Institute of Polish Academy of Sciences, Nicolaus Copernicus University, 01-447b Warsaw, Poland.

## Abstract

Brain–computer interface (BCI) technology, which controls external devices by directly decoding brain activities, has made important progress and practical applications in recent years in many fields. However, the domain bias issue in cross-domain applications remains a significant challenge in the practical implementation of BCI technology. This is particularly acute in scenarios where target data are unavailable, largely because of the noise sensitivity and acquisition limitations inherent in electroencephalography (EEG) signal data. When processing nonstationary EEG signals, existing domain generalization methods face limitations: Adversarial training may compromise model stability, while global feature alignment approaches struggle to sufficiently decouple category-dependent and category-independent features, thereby constraining generalization performance. Therefore, in this paper, we propose a hybrid approach based on domain-invariant feature learning and data enhancement. We introduce a “fixed” structure enhancement method that combines domain-invariant feature learning with data enhancement strategies, decouples domain-invariant features from other features, optimizes cross-domain feature extraction, and reduces the effect of noise in data. Through extensive experimental validation on multiple publicly available datasets, the model proposed in this paper outperforms the existing state-of-the-art methods, providing a novel and effective solution to the domain bias problem in BCI.

## Introduction

In recent years, with the rapid development of intelligent hardware and intelligent algorithms, brain–computer interface (BCI) technology has made significant progress and has been widely used in various fields [1–3]. Despite the many advances in BCI technology, practical applications still face the generalizability problem, especially in cross-domain applications, where the domain bias problem poses a significant challenge to model performance [4]. Traditional BCI decoding methods typically assume that data follow an independent and identically distributed model, meaning that training and testing data originate from the same distribution [5]. However, because of individual differences, device variations, and changes in experimental environments, electroencephalography (EEG) signals may exhibit significant distributional discrepancies between training and testing data [6], which can severely degrade model performance on unseen subjects.

In recent years, deep learning has shown great potential in several domains [7]. Deep-learning-based domain adaptation (DA) and domain generalization (DG) techniques have been proposed to enhance model performance across different domains by learning the relationships between the source domain and target domain [8,9]. However, in practice, the application of DA methods based on target data is limited because of the scarcity and inaccessibility of target data [10].

Unlike DA, DG aims to perform effective inference in unseen target domains by learning a generalized model from multiple source domains. Instead of relying on the target domain data, DG learns a generic representation model for cross-DG directly from the source domain data [11].

Existing DG methods can be mainly classified into 3 categories: techniques such as data augmentation [12,13], domain-invariant feature learning [14], and meta-learning [15,16], which have achieved rapid development in areas such as computer vision and natural language processing. However, most of these methods focus on specific application scenarios, and relatively little research has been conducted in the field of EEG.

In the domain of motor imagery (MI), DGMA combines data augmentation strategies with adversarial learning methods to minimize domain differences, achieving domain transferability and recognizability [17]. Subsequently, researchers integrated meta-learning with maximum mean discrepancy (MMD) strategies to align cross-domain features [18]. Supervised contrastive learning-based domain generalization network (SCLDGN), meanwhile, learns domain-invariant features using a domain alignment mechanism to constrain cross-domain representation distances [4].

Although DG offers novel approaches to addressing the issue of data invisibility in target domains, numerous challenges persist in applying DG methods to BCI due to the high dimensionality, nonstationarity, and interindividual variability of EEG signals. First, data augmentation methods, lacking explicit optimization objectives, may introduce irrelevant noise while enhancing data diversity. Adversarial learning strategies are often destabilized by nonstationary EEG signals during model training. Second, feature alignment techniques fail to fully decouple fine-grained category features through global statistics. Moreover, they are insensitive to the covariance structure of multisource heterogeneous EEG signals, which may potentially suppress task-discriminative neural patterns. Meta-learning imposes strict computational complexity and task design requirements, limiting its application in high-dimensional, nonstationary EEG data. How to effectively solve these problems and improve the robustness of models across domains and tasks has become the key to promoting the practical application of BCI technology.

Recently, in the field of computer vision, methods that explicitly partition features into categories such as internal invariants and mutual invariants to enhance model generalization have demonstrated significant potential [19,20]. However, most of these approaches lack specialized mechanisms for handling the unique spatiotemporal characteristics of EEG data, resulting in critical limitations when directly applied to brain signals. Lu et al. [19] defines its intrainvariant features based on a predefined Fourier phase. However, as a general signal processing concept, Fourier phase may not optimally capture the neural semantic information most relevant to MI tasks. Furthermore, its feature guidance via a knowledge distillation framework and simple feature segmentation strategy may fail to achieve effective and thorough decoupling of category-related semantics from domain-specific noise in EEG signals. This increases model complexity and limits its robustness in cross-subject EEG decoding.

Therefore, drawing inspiration from the concepts of domain-invariant and domain-variant features in image processing and considering the characteristics of EEG signals, we introduce the idea of feature decoupling into the MI task. By designing a fixed structure decoupler and integrating structured feature learning with customized data augmentation strategies, we construct a DG model focused on invariant feature extraction (DGIFE).

This model addresses 2 primary challenges in EEG signal processing: First, existing DG methods typically rely on global domain alignment, yet such approaches struggle to uncover sufficiently diverse and robust invariant feature sets, resulting in limited adaptability to intersubject nonstationary covariance characteristics. Second, while traditional data augmentation strategies can expand sample size, they readily introduce irrelevant noise that significantly affects EEG signals and often overlook domain-specific bias effects, thereby limiting the effectiveness of augmentation.

The method we developed requires no predefined feature transformations. It dynamically and adaptively separates category-relevant and irrelevant features from raw EEG signals in an end-to-end manner, guided by a comprehensive exploration and alignment loss. This design enables the model to autonomously discover the most discriminative and generalizable feature combinations, offering a more direct and effective approach to addressing domain bias in BCI.

Furthermore, leveraging the distinct spectral–spatial patterns across MI categories, we designed a data augmentation method distinct from simple dataset expansion. A fine-grained patch strategy simulates diverse frequency band information to capture fine-grained EEG spectral features. Subsequently, to further decouple fine-grained category features, we combined gating and attention mechanisms to dynamically capture intersubject variability in brain activity space at finer granularity, extracting spectral–spatial patterns. Finally, to address the high intraclass variance in EEG, we introduce the increase intraclass spacing (interclass prototype network [IPN]) module. This module actively enforces the principles of “intraclass compactness” and “interclass separation”, ensuring that the decoupled features are not only domain invariant but also highly discriminative. Under noise constraints, angle optimization is achieved, making the approach more robust to noise and domain shifts.

Through theoretical analysis and experimental validation, this paper demonstrates the superiority of the method in the task of MI classification and compares it with existing methods to prove the advantages of the proposed model. In conclusion, the main contributions of this paper can be summarized as follows:

1. We propose a data-driven structural decoupling framework and design a fixed structure-augmented decoupler that actively learns and separates task-relevant domain-invariant features in an end-to-end manner.
2. The IPN module is introduced to perform deep structural optimization on the decoupled feature space, ensuring that the learned domain-invariant features possess high class discriminability.
3. We constructed an end-to-end DG model specifically tailored for EEG signals, extracting key spatiotemporal features of motor intent through fine-grained temporal encoding and gated attention mechanisms. Extensive experiments demonstrate that our approach significantly outperforms baseline models and state-of-the-art methods, while providing plausible explanations consistent with neural mechanisms through feature visualization.

## Materials and Methods

### DGIFE model

**Fig. 1.**
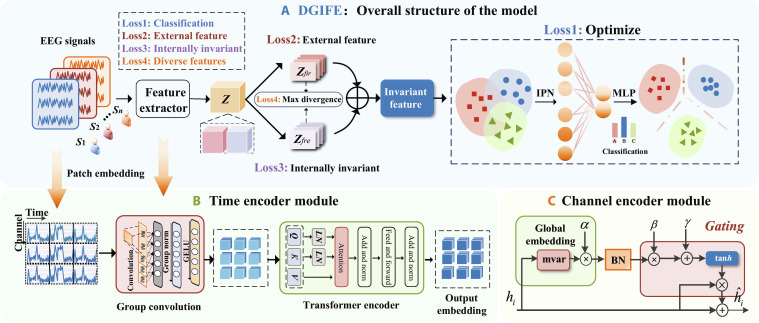
Model structure. (A) Overall structure of the model. MLP, multilayer perceptron. (B) Structure of the time encoder module. (C) Structure of the channel encoder module. BN, batch normalization.

Our proposed DGIFE model consists of 3 main modules as shown in Fig. 1. The architecture consists of 2 core parts: the feature extractor module and the domain-invariant feature extraction module. The feature extractor module consists of 2 parts. First, fine-grained EEG features in multiple frequency bands are extracted by a patch strategy to enrich the available information. Then, a channel encoding module is used to highlight important spatial EEG channels. The domain-invariant feature extraction module divides the features extracted by the feature extractor into 2 parts, prompting the network to learn the internal features and the external features, while the IPN is introduced to optimize the feature space and make classification decisions. Next, we describe the specific implementation of each module in detail.

#### Time encoder module

The raw EEG signal $X\in {\mathbb{R}}^{C\times T}$ comprises data from *C* electrodes across *T* time points. Instead of traditional methods such as up/downsampling or manual band filtering, we use a fine-grained patch segmentation method with different sampling frequencies, which enables us to simulate different frequency bands and efficiently capture the features associated with the bands. The raw EEG signal *X* is segmented into N patch segments, each of length $L_{s}=125$. The cut gives $\begin{array}{c} x=\left\{x_{p_{i_{j}}\;,k}\in R^{L_{s}}\left|j=1,2\ldots ,C,k=1,2\ldots ,N=\left[\frac{T}{L_{s}}\right]\right.\;\right\} \end{array}$*.* $p_{i_{j}}$ denotes the data at the corresponding time point for channel *j* in the *k*th patch. Specifically, we use a time-domain convolutional module for temporal feature extraction, comprising a 2-dimensional convolutional layer, a normalization layer, and a Gaussian error linear unit (GELU) activation function [21]. The 3 convolutional kernels have dimensions of (1,64), (1,32), and (1,16), respectively. The multigranularity output patch embedding is denoted as shown in Eq. (1):

$$x_{n}=\left\{e_{p_{i_{j}}\;,k}\in R^{D}\left|j=1,2,\ldots ,C,k=1,2,\ldots ,\left[\frac{T}{L_{s}}\right]\right.\;\right\}$$ (1)

To improve the stability and efficiency of fine-grained EEG feature training, we introduce a self-attention mechanism to further extract and fuse the features, as shown in Eq. (2):

$$\mathrm{Att}\left(Q,K,V\right)=\text{softmax}\left(\frac{\mathrm{LN}\left(Q\right)\mathrm{LN}\left(K\right)}{\sqrt{d_{k}}}\right)V$$ (2)

where $d_{k}$ is the dimension of a head in a multihead note and *LN* denotes the layer regularization layer. *Q*, *K*, and *V* are derived from the input $x_{n}$ through independent learnable linear projection weights $Q=W_{Q}x_{n}$, $K=W_{k}x_{n}$, $V=W_{v}x_{n}$, where $W_{Q}$, $W_{k}$*,* $\;W_{v}$ are the learnable projection matrix and the head in self-attention is 1.

Subsequently, a channel attention module is introduced to extract EEG spatial features, the core idea is to take into account that different categories have different activation patterns in the spectral space domain.

#### Channel encoder module

Inspired by gated channel switching [22], we introduce a gating mechanism to adjust the information flow between channels and thus construct a spatial spectrum attention module. This module dynamically adjusts the distribution of different channels by calculating the attention weights of each channel, thus focusing attention on channels that exhibit significant activation patterns in different tasks. The realization formula of spatial spectral attention is shown in Eqs. (3) and (4).

$$x_{t}=\text{reshape}\left(x_{n}\right),\;x_{t}\in {\mathbb{R}}^{C\times \left(N\times D\right)}$$ (3)

$$s=\alpha \cdot \text{mvar}\left(x_{t}\right)$$ (4)

The output of the time encoder module is $x_{n}\in {\mathbb{R}}^{\left(C\times N\right)\times D}$, we use a reshape strategy to rearrange the input features and separate the channel dimension. A channel normalization method is then used to model the channel relationships, as shown in Eq. (5):

$$\hat{s\;}=\frac{\sqrt{C}s}{{\left\|s\right\|}_{2}}=\frac{\sqrt{C}s}{{\left[\left(\sum_{c=1}^{C}s^{2}\right)+\epsilon \right]}^{1/2}}$$ (5)

where *ϵ* = 1 × 10^−5^ is a small constant to avoid derivation problems at zero. The gating weights and biases *γ* and *β* are responsible for scaling the input features by channel direction, as shown in Eqs. (6) and (7).

$$\mathrm{att}=1+\tan h\left(\beta \hat{s}+\gamma \right)$$ (6)

$$x_{\mathrm{ch}}=\mathrm{att}x_{t}$$ (7)

The $x_{\mathrm{ch}}\in {\mathbb{R}}^{C\times \left(N\times D\right)}$ output by the channel attention module assigns different weights to each channel. *γ* and *β* are obtained through random initialization. After passing through the channel attention module, the EEG signal feature are better fused. To effectively capture the multiscale temporal domain features of the input signal, we introduce a multiscale pooling module. This module achieves this by segmenting the input feature map along the channel dimension to obtain $x_{\text{split}}=\text{split}\left(x,\frac{d}{L},\dim=1\right)$, where *L* = 3. Each segmented feature is fed into 3 parallel pooling paths for processing. Each path uses distinct pooling sizes (25, 50, and 100 pixels) and utilizes variance pooling to extract feature representations. Finally, the outputs from each path are flattened and merged to aggregate multiscale information. Ultimately, the feature vector output from the pooling layer is *Z* = {*z*_1_, *z*_2_, …, *z_i_*}, where *i* = 1, 2, …, *m*.

#### Domain-invariant feature

To explicitly decouple EEG features, we abandoned implicit methods reliant on complex variational inference or adversarial training and instead introduced a fixed structure decoupler with explicit inductive bias. This decoupler physically decomposes feature vectors into complementary class-related feature ($z_{\mathrm{fre}}$) and class-independent feature ($z_{\mathrm{fir}}$) streams, directly distinguishing task-related neurophysiological patterns—event-related desynchronization (ERD) and event-related synchronization (ERS)—from task-irrelevant variations and noise. By integrating multiloss supervision, the method effectively purifies category-dependent features, rendering them robust against confounding factors and thereby enhancing model generalization.

Our structural decoupling mechanism achieves effective feature decoupling through the synergistic interaction of multiple loss functions. The optimization process forms a virtuous cycle: First, $L_{\text{align}}$ injects domain-specific information into $z_{\mathrm{fir}}$. Then, $L_{\exp}$ ensures that this information does not remix into $z_{\mathrm{fre}}$. Finally, $L_{\mathrm{cls}}$ further guarantees that $z_{\mathrm{fre}}$ maintains task-specific discrimination capabilities. The synergistic interaction of these distinct losses provides an enabling framework for explicit separation.

Let z be the features obtained after the feature extractor. Then, they are divided into 2 features with the same size: the class-related feature $z_{\mathrm{fre}}$ and the class-independent feature $z_{\mathrm{fir}}$, The *λ* parameter in our model serves as a balancing coefficient to control the relative weights of different loss terms within the overall optimization objective λ∈ [0,1]. We set *λ* = 0.5 to emphasize category-related features while preserving useful background information. as shown in Eq. (8):

$$z=\lambda z_{\mathrm{fre}}+\left(1-\lambda \right)z_{\mathrm{fir}}$$ (8)

However, only a simple feature splitting operation is not enough to fully realize the independence of these 2 types of features because, in practical applications, there are often certain intersection and interdependence between $z_{\mathrm{fre}}$ and $z_{\mathrm{fir}}$. Simple decoupling methods may not be able to eliminate such dependencies, so more complex mechanisms are needed to further realize feature independence.

To address this problem, we further enhance the separability of $z_{\mathrm{fre}}$ and $z_{\mathrm{fir}}$ by designing and introducing different loss functions as constraints. In EEG signal analysis, the covariance structure between signals across different channels encodes crucial information about functional connectivity within the brain, effectively capturing the collaborative characteristics between left and right brain regions during MI. Compared to traditional MMD loss, which may be overly sensitive to global amplitude shifts caused by variations in recording conditions when matching overall distributions, our covariance-based alignment method directly operates on second-order statistics. This enables more robust capture and matching of task-related spatial collaboration patterns, as demonstrated in Eq. (9):

$$L_{\text{align}}=\frac{2}{N\left(N-1\right)}\sum_{i\neq j}^{N}{\left\|c_{i}-c_{j}\right\|}_{F}^{2}$$ (9)

where $c_{i}=\frac{1}{n_{i}-1}\left(z_{i}^{T}z_{i}-\frac{1}{n_{i}}{\left(1^{T}z_{i}\right)}^{T}\left(1^{T}z_{i}\right)\right)$ is the covariance matrix and $\left\|\cdot \right\|$ is the matrix Frobenius norm.

Since there are some duplicated and redundant information between class-related features and class-independent features, we hope that these 2 parts of the features can be extracted as much as possible to complement each other and avoid the extraction of duplicate features. The diversity of the proposed features can also be further enriched by this approach, thus improving the generalization ability of the model.

To achieve this objective, we minimize the loss function $L_{\exp}\left(z_{\mathrm{fre}},z_{\mathrm{fir}}\right)$ to maximize the distance between $z_{\mathrm{fre}}$ and $z_{\mathrm{fir}}$ in the feature space. This operation aims to enhance the independence between the 2 types of features, preventing mutual interference during representation learning. Its specific definition is shown in Eq. (10).

$$L_{\exp}\left(z_{\mathrm{fre}},z_{\mathrm{fir}}\right)=-d\left(z_{\mathrm{fre}},z_{\mathrm{fir}}\right)$$ (10)

where $d\left(\cdot ,\cdot \right)$ is a distance function, and we simply use the L2 distance.

Assuming that there exist *N* source domains ${\left\{s_{i}\right\}}_{i=1}^{N}$, the data distribution of each source domain is $p_{i}\left(x,y\right)$, the distribution of the target *T* is unknown, and the goal of the model is to learn the mapping function f:x→y, such that the expected risk is minimized over the target domain $R_{T}\left(f\right)\leq E_{x,y∼T}\left[l\left(f\left(x\right),y\right)\right]$. We determine the upper limit of the minimum generalized error according to [23], as shown in Eq. (11):

$$\epsilon_{t}\leq \epsilon_{s}+d_{H}\left({\mathbb{P}}_{s},{\mathbb{P}}_{t}\right)+O\left(\sqrt{\frac{\log\;d}{n}}\right)+\lambda_{H}$$ (11)

where $\epsilon_{s}$ is the source domain empirical error, $d_{H}\left({\mathbb{P}}_{s},{\mathbb{P}}_{t}\right)$ is the domain difference term under the assumption of H space, and $O\left(\sqrt{\frac{\log\;d}{n}}\right)$ is the model complexity term. Here, covariance alignment is performed on multisource target data by $d_{H}\left({\mathbb{P}}_{s},{\mathbb{P}}_{t}\right)$ as shown in Eq. (12). This heuristic design, based on covariance alignment, effectively reduces differences in second-order statistics. The Rademacher complexity of the DGIFE method is constrained by the feature dimension, as shown in Eq. (13). On the basis of the above analysis, the upper bound of the objective domain error for the DGIFE method can be approximated as expressed in Eq. (14).

$$d_{H}\left(P_{s},P_{t}\right)\approx \sqrt{\frac{1}{S}\sum_{s=1}^{S}{\left\|C_{s}-C_{t}\right\|}_{F}^{2}}$$ (12)

$$R\left(K_{\text{DGIFE}}\right)\leq \sqrt{\frac{\log\;d_{\mathrm{fre}}+\log\;d_{\mathrm{fir}}}{n}}$$ (13)

$$\epsilon_{t}^{\text{DGIFE}}\leq \epsilon_{s}^{\text{DGIFE}}+\sqrt{\frac{1}{S}\sum_{s=1}^{S}{\left\|C_{s}-C_{t}\right\|}_{F}^{2}}+\sqrt{\frac{\log\;d_{\mathrm{fre}}+\log\;d_{\mathrm{fir}}}{n}}+\lambda_{H}$$ (14)

The traditional single-domain alignment method MMD has an upper bound on the error as shown in Eq. (16):

$$\epsilon_{t}^{\mathrm{MMD}}\leq \epsilon_{s}^{\mathrm{MMD}}+\sqrt{\mathrm{MM}D^{2}\left({\mathbb{P}}_{s},{\mathbb{P}}_{t}\right)}+O\left(\sqrt{\frac{\log\;d}{n}}\right)+\lambda_{H}$$ (15)

MMD2Ps,Pt=Ex,x′~Pskx,x′+Ey,y′~Ptky,y′−2Ex~Ps,y~Ptkx,y (16)

where *k*(·,·) is the kernel function, for the Gaussian kernel $k\left(x,y\right)=\exp\left(-\gamma {\left\|x-y\right\|}^{2}\right)$, whose second-order Taylor expansion is shown in Eqs. (18) and (19).

$$\mathrm{MM}D^{2}\approx \gamma^{2}\left({\left\|\mu_{s}-\mu_{t}\right\|}^{2}+\mathrm{Tr}\left(C_{s}+C_{t}-2C_{s}^{\frac{1}{2}}C_{t}^{\frac{1}{2}}\right)\right.$$ (17)

$$\begin{array}{c} \begin{array}{c} \begin{array}{c} \begin{array}{c} \epsilon_{t}^{\mathrm{MMD}}\leq \epsilon_{s}^{\mathrm{MMD}}\\ +\sqrt{\gamma^{2}\left({\left\|\mu_{s}-\mu_{t}\right\|}^{2}+\mathrm{Tr}\left(C_{s}+C_{t}-2C_{s}^{\frac{1}{2}}C_{t}^{\frac{1}{2}}\right)\right.}+O\left(\sqrt{\frac{\log\;d}{n}}\right)+\lambda_{H}\; \end{array} \end{array} \end{array} \end{array}$$ (18)

When the source and target domain covariance matrices $C_{s}\neq C_{t}$, the sensitivity of MMD to covariance is dominated by $\mathrm{Tr}\left(C_{s}+C_{t}-2C_{s}^{\frac{1}{2}}C_{t}^{\frac{1}{2}}\right)$, and due to the smoothing of the kernel function, the response to covariance differences of this trace term exhibits a sublinear growth.

The covariance difference is directly amplified by the Frobenius paradigm, which satisfies $\frac{\partial L_{\text{align}}}{\partial {\left\|C_{s}-C_{t}\right\|}_{F}}\propto {\left\|C_{s}-C_{t}\right\|}_{F}$, and has higher gradient efficiency compared to the sublinear response of MMD. In addition, the sparse interaction, combined with regularized, forced covariance matrix, can avoid redundant dimensional alignment. Compared to traditional MMD methods, this approach simultaneously controls model complexity within the structured feature space through direct covariance alignment and structured feature control, thereby promising to achieve superior generalization performance.

#### Increase intraclass spacing

Recent studies have shown that cross-entropy loss (CE) has limited effectiveness in reducing within-class variance [24], especially in the case of EEG signals, where CE loss is difficult to effectively handle signal fluctuations and variance due to its inherent nonstationarity. This leads to a loose distribution of sample features near the decision boundary, which does not capture the compactness of intraclass features well. To overcome this problem, we introduce prototype learning. Prototype learning not only pushes the sample features to the category prototypes through classification loss but also further optimizes the sample features through prototype loss [25], which promotes the tightness between the sample features and the prototypes, thus enhancing the tightness within the categories.

Because of significant variability in EEG signal amplitude across different experiments, we use a cosine-based loss function, which makes the model focus more on the angle between vectors than on the mode length, which helps improve the model’s robustness and generalization. The loss shown in Eq. (19) demonstrates the growth of normalized prototype vectors by introducing a scaling factor *t* to control the cosine distance.

The IPN vectors are initialized on the basis of class centers during the early training phase. Subsequently, they are updated by minimizing the cosine classification loss alongside the network parameters, as shown in Eq. (20). This maximizes the similarity between samples and prototypes, thereby reducing intraclass variance. The temperature scaling factor *t* introduces interclass competition effects into the prototype network through the softmax normalization mechanism. When sample features converge toward the correct prototype, this mechanism effectively increases the angular distance between prototypes of different classes.

Specifically, *t* influences feature learning through 2 mechanisms: Higher *t* values amplify gradient signals during backpropagation, making the model more sensitive to subtle angular differences between features and prototypes, thereby promoting more precise feature alignment. As *t* increases, the probability distribution of softmax outputs becomes more peaked, amplifying confidence differences between the predicted class and others and strengthening the decision boundary. In the experiment, we conducted experiments with different experience values and selected the appropriate *t* value. Our IPN model defines prototypes as learnable parameters, enabling dynamic optimization of the feature space. Its cosine similarity metric demonstrates greater robustness to amplitude-varying EEG signals, while the multitask learning mechanism effectively prevents prototype contamination by noise.

$$L_{\mathrm{cls}}=-\frac{1}{m}\sum_{i=1}^{m}\log\frac{e^{t\cos\theta_{y_{i}}^{\left(i\right)}}}{\sum_{j=1}^{n}e^{t\text{cos}\theta_{j}^{\left(i\right)}}},\cos\theta_{j}^{\left(i\right)}=\frac{s_{j}\cdot z_{i}}{S{\left\|z_{i}\right\|}_{2}}$$ (19)

$$\frac{\partial L_{\mathrm{cls}}}{\partial_{s_{j}}}=t\left(\delta_{j=y_{i}}-P_{j}\right)\cdot \frac{z_{i}}{{\left\|z_{i}\right\|}_{2}}$$ (20)

where *m* denotes the number of samples, *n* denotes the number of categories, $z_{i}$ denotes the feature that is the *i*th sample, $y_{i}$ denotes the label of the *i*th sample, *s* denotes the IPN, and $s_{j}\in {\mathbb{R}}^{d}$ is the IPN of the *j*th class. Minimizing $L_{s}$ facilitates the separation of the features from the different classes. $P_{j}$ is the softmax probability, and δ is the indicator function.

Because of the nature of the soft algorithm [26], we ensure that the training converges to a constant value by limiting the number of paradigms of the IPN to a small value, ${\left\|s_{i}\right\|}_{2}\leq S$ (weights normalized) to expand the distance from the feature vector to the origin for better interclass classification.

In summary, the final optimized objective of the proposed method in this paper is as follows:

$$\begin{array}{c} \begin{array}{c} \min E_{{\left(x,y\right)}^{∼}}L_{\mathrm{cls}}\left(G_{c}\left(G_{f}\left(x_{i}\right)\right),y\right)\\ +\lambda_{1}L_{\mathrm{mse}}\left(z_{\mathrm{fre}},G_{f}\left(x_{i}\right)\right)+\lambda_{2}L_{\text{align}}+\lambda_{3}L_{\exp}\left(z_{\mathrm{fre}},z_{\mathrm{fir}}\right) \end{array} \end{array}$$ (21)

where $G_{c}$ and $G_{f}$ are the classification layer and feature network respectively. $\lambda_{1}$, $\lambda_{2}$, and $\lambda_{3}$ are hyperparameters representing the weight values of loss1, loss2, and loss3 respectively. The formulation contains 3 objectives: classification, internal invariant feature learning, and external feature learning. In the DG task, the first and third terms are the 2 common goals of DG invariant representation learning. However, existing research has shown that relying on these 2 goals alone is not sufficient to address the challenges in cross-domain applications. By simultaneously exploring diverse features (including the second loss and the last loss), we can effectively alleviate this problem and thus improve the performance of the model, and subsequent experimental results validate the effectiveness of this approach. The detailed training procedure is described in Algorithm 1.


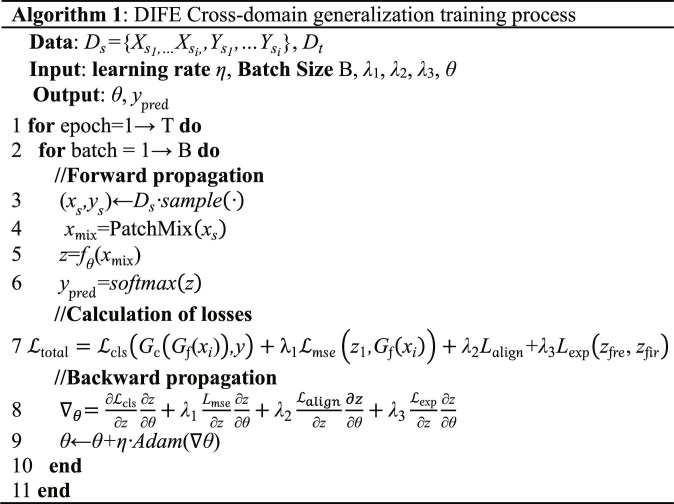


### Dataset

• Dataset I: Giga dataset [27] contains MI data from a total of 52 healthy subjects, including 33 males and 19 females. The tasks performed by the subjects were imagining left- and right-hand movements based on a screen. Each subject completed a total of 200 or 240 experiments. The number of electrodes used in the experiment is 64.
• Dataset II: The OpenBMI dataset [28] includes MI data from 54 healthy subjects, including 25 females and 29 males. The task performed by each subject was to imagine a specified movement for the left and right hands based on left and right arrow cues on the screen. Each subject completed 200 trials. The number of electrodes used for the experiment is 62.
• Dataset III: The BCIC-IV-2a dataset [29] contains MI data from 9 healthy subjects. The task performed by each subject was to imagine 4 movements. Each subject completed 288 experiments, which used a number of 22 electrodes.

For all datasets, we extracted EEG data within a 0- to 4-s time window following the presentation of visual cues to cover the ERD phase during MI execution. All data were resampled to 250 Hz. Subsequently, a fourth-order Butterworth bandpass filter (4 to 40 Hz) was applied to preserve rhythm characteristics associated with MI while suppressing low-frequency drift and high-frequency noise. In addition, to mitigate the impact of artifacts, trials with voltage amplitudes exceeding ±100 μV were excluded.

#### Experimental setup

To rigorously evaluate the validity and generalizability of the proposed DGIFE model in cross-subject scenarios, we use the leave-one-subject-out (LOSO) principle. In each LOSO fold, we select one subject as the test set, while data from all the remaining subjects are used for the training set. It is important to note that the different session data from each subject are merged into one overall set. This approach fully takes into account the problems of domain bias and session bias faced by EEG-based BCI applications in practice. Ultimately, in all experiments, we report the average of the performance of external tests across disciplines to fully evaluate the generalization ability and robustness of the model. The model was trained using the Adam optimizer with an initial learning rate of 0.001. Trained with a batch size of 128, the proposed DGIFE model was implemented using Python 3.7 and performed on NVIDIA GeForce RTX 3090 GPUs using PyTorch 1.10.

## Results

To validate the effectiveness of the proposed DGIFE model, we compare the model proposed in this paper with representative methods: EEGNet [30], Shallow ConvNet [31], Deep ConvNet [32], InterpretableCNN [33], ATCNet [34], SST-DPN [35], MSVTNet [36], DBConformer [37], and SCLDGN [4]. To further validate the effectiveness of the proposed DGIFE method, we compare it with strong universal generalization baseline models Mixup [38], DIFEX [19], and URM [20]. The experimental results are shown in Table 1.

**Table 1.** Experimental results compared to other models, where the bold values indicate the best experimental results

Methods	Dataset I		Dataset II		Dataset III	
	ACC/%	SD	ACC/%	SD	ACC/%	SD
EEGNet [30]	72.02	12.07	78.28	9.51	61.67	10.17
Shallow ConvNet [31]	74.13	12.11	81.38	8.54	60.63	16.87
Deep ConvNet [32]	71.66	12.26	82.01	**7.98**	59.49	9.56
InterpretableCNN [33]	58.29	15.43	68.65	10.14	57.89	14.54
ATCNet [34]	70.46	12.33	77.44	9.41	60.72	10.03
SST-DPN [35]	74.32	10.47	82.06	9.56	59.73	10.25
MSVTNet [36]	70.31	12.90	78.48	9.52	62.13	12.16
DBConformer [37]	56.90	15.19	65.10	10.49	54.36	9.89
SCLDGN [4]	68.34	10.54	81.58	9.36	63.83	9.57
Mixup [38]	73.28	12.01	67.15	8.20	38.77	10.24
DIFEX [19]	64.25	11.14	63.04	8.93	27.08	12.88
URM [20]	70.50	13.33	68.79	9.90	31.36	23.76
**DGIFE**	**77.36**	**10.40**	**84.08**	8.96	**64.74**	**9.13**

### Analysis of experimental results

Table 1 lists the average classification accuracies achieved by the different algorithms on the evaluation datasets. In terms of accuracy (ACC), the DGIFE model achieves better experimental results on 3 datasets. This shows that the DGIFE model can effectively decode EEG signals, and its overall performance is better than most of the comparison models. The standard deviation (SD) reflects the stability of the model, and the SD values of the DGIFE model are relatively small in the 4 datasets, such as 10.40 in dataset I and 8.96 in dataset II, which indicates that the performance fluctuation of the DGIFE model is small in different test samples.

We believe that the superiority of our model over comparable approaches stems from the targeted design of its key components, which directly address core challenges faced by the MI paradigm in DG. First, the explicit feature decoupling mechanism structurally separates input signals into category-dependent and -independent streams, enabling directional noise reduction. This allows the model to focus on ERD/ERS neural responses closely related to MI, thereby enhancing feature robustness and generalization capabilities. Second, addressing the temporal characteristics of MI tasks, our fine-grained temporal encoder effectively captures multiband temporal patterns. Concurrently, the gated channel attention mechanism focuses on MI-relevant key brain regions, improving spatial specificity of features and better adapting to the nonstationary nature of EEG signals. Finally, the introduced IPN module significantly enhances feature discriminability by optimizing category prototypes and decision boundaries. Synergizing with the exploration loss, it effectively reduces intraclass variance and increases interclass distance, thereby maintaining stable classification performance on unseen subjects.

Compared to generic DG methods, the approach presented in this paper underscores the necessity of domain-specific design. While these generic methods perform well in computer vision, they fail to achieve optimal performance on EEG data. By integrating components such as fine-grained temporal encoding and gated attention, DGIFE can specifically address the unique challenges of EEG signals, whereas generic methods lack effective modeling capabilities in such scenarios.

### Ablation experiments

To further analyze the effects of the multigranularity patch coding module and the channel attention coding module on the overall performance of the model, we conduct ablation experiments on 3 datasets. We design 2 variant models for ablation studies: DGIFE (without patch [w/o patch]): this variant removes the patch-based fine-grained time encoder to demonstrate its importance; DGIFE (without channel [w/o channel]): this variant removes the gated channel attention encoder to demonstrate its importance.

**Fig. 2.**
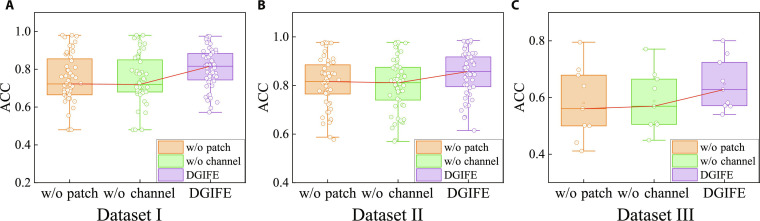
Ablation experimental results on the datasets. (A) Dataset I. (B) Dataset II. (C) Dataset III.

From Fig. 2 and Table 2, it can be seen that the average ACC of the models on all datasets is lower after removing the fine-grained coding module, with 12.88 for the patch model SD and 10.40 for the DGIFE model in dataset I. This implies that the absence of the multigranular patch coding module leads to increased fluctuations in the model performance and poorer stability across different test samples, which further suggests that the fine-grained coding module is capable of extracting diversified features of the EEG signals at different scales, which effectively enhances the model’s adaptability to the data and its ability to generalize.

**Table 2.** Results of ablation experiments, where the bold values indicate the best experimental result

Methods	Dataset I		Dataset II		Dataset III	
	ACC/%	SD	ACC/%	SD	ACC/%	SD
w/o patch	73.69	12.88	81.02	10.10	60.08	12.21
w/o channel	73.85	12.53	80.27	10.54	60.76	9.94
**DGIFE**	**77.36**	**10.40**	**84.08**	**8.96**	**64.74**	**9.13**

After removing the channel attention module, the performance of the model on each dataset is similar to that of removing the fine-grained coding module, indicating the importance of the channel attention module for extracting effective features, which is capable of focusing on the most informative parts of complex data by assigning appropriate attention weights to different channel features.

### Exploring the effect of different losses on the model

Subsequently, to comprehensively assess the effects of different loss functions in our proposed DGIFE model on the experimental results, we explored the effects of different kinds of loss functions on the model separately. Specifically, we designed the following 4 different models: (a) replacement of loss1: No IPN network is introduced, and softmax and CE networks are directly utilized for model training and classification; (b) without loss2: Loss2 encourages the model to learn more invariant features by minimizing the correlation between invariant and coded features; (c) without loss3: The loss function loss3 is removed without the alignment of decoupled features to evaluate the performance of the model; (d) without loss4: No constraints on the distances between internal and invariant features are imposed.

• Replacement loss1: As can be seen from Table 3, the performance is lower than the DGIFE model on all datasets. The ACC in dataset I is 73.32%, which is about 4 percentage points lower than that of DGIFE model, and the SD is as high as 12.79. The IPN network helps improve the model’s ability to differentiate when dealing with complex data and thus exhibits stronger performance in cross-task and cross-domain decoding tasks.
• Loss2: The ACC and stability of the models without loss2 varies in datasets I to III. If this loss function is removed, the model will lose the effective learning of invariant features, and the performance of the model will be significantly degraded especially when dealing with data with large domain differences. Therefore, the presence of loss2 provides the model with the necessary regularization constraints that enable the model to be more robust and maintain high ACC across different datasets and tasks.
• Loss3: By removing loss3, the performance on each dataset is also affected. The experimental results show that the role of loss3 in feature decoupling and alignment should not be neglected. Without this loss function, the model cannot effectively distinguish between domain-invariant features and task-specific features, which not only hinders the efficient extraction of features but also leads to overfitting of the model in the face of variable data distributions.
• Loss4: Without the constraint on the distance between domain-invariant features and other features, the ACC of 76.58% is close to that of the DGIFE model in dataset I, but there is still a gap in other datasets, such as dataset II. Without this constraint, the model tends to suffer from overfitting some specific features, resulting in limited diversity of feature extraction, which reduces the decoding ACC of the model.

**Table 3.** Exploring the effect of different losses on the model, where the bold values indicate the best experimental results

Methods	Dataset I		Dataset II		Dataset III	
	ACC/%	SD	ACC/%	SD	ACC/%	SD
Loos1	73.32	12.79	82.61	9.81	59.93	9.89
Loos2	75.71	11.13	82.46	10.03	62.04	9.39
Loss3	75.56	12.39	81.90	9.65	62.98	10.27
Loss4	76.58	11.34	83.38	9.50	63.52	9.93
**DGIFE**	**77.36**	**10.40**	**84.08**	**8.96**	**64.74**	9.13

### Parametric sensitivity

**Fig. 3.**
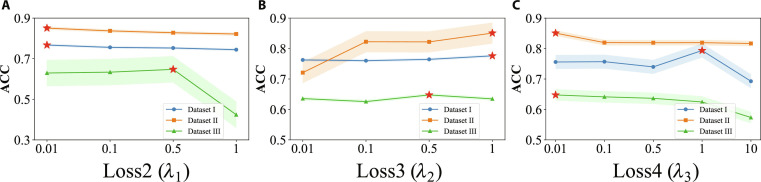
Experimental results with different parameters. (A) Loss2 (*λ*_1_). (B) Loss3 (*λ*_2_). (C) Loss4 (*λ*_3_).

The method proposed in this paper has 3 main hyperparameters: $\lambda_{1}$for extracting invariant features, $\lambda_{2}$ for aligning losses, and $\lambda_{3}$ for increasing decoupled feature distance pairs. We evaluate the parameter sensitivity of our method in Fig. 3, where we vary one parameter and fix another to record the results.

As can be seen through the experimental results from these results, the ACC slightly decreases when the value of the $\lambda_{1}$ parameter is varied from 0.01 to 1. When the parameter $\lambda_{1}$ is taken to be 1, the overall fluctuation on the 3 datasets is less overall. $\lambda_{2}$ performs optimally with $\lambda_{2}$ of 1 in datasets I to III. These results indicate that different datasets have different sensitivities to hyperparameters, and choosing appropriate hyperparameters can significantly improve model performance.

To investigate the impact of the temperature coefficient on model performance, we systematically evaluated the IPN module’s behavior under different temperature settings, with results shown in Table 4. Experiments demonstrate that the value of the temperature coefficient *t* significantly affects classification performance: When *t* = 5, the model achieved superior results, balancing feature distribution sharpening with gradient stability to yield the best generalization performance. Both excessively high and low *t* values lead to performance degradation, highlighting the critical role of temperature scheduling in prototype learning.

**Table 4.** Exploring the effect of different losses on the model, where the bold values indicate the best experimental results

Methods	Dataset I		Dataset II		Dataset III	
	ACC/%	SD	ACC/%	SD	ACC/%	SD
0.5	71.46	13.99	82.03	56.75	63.47	8.75
1	72.68	14.04	83.78	10.02	**64.74**	9.13
5	**77.36**	**10.40**	**84.08**	**8.96**	64.14	9.45
10	74.66	19.71	81.54	12.31	31.21	**4.52**

### Robustness analysis against noise

We quantitatively evaluate the noise robustness of the proposed DGIFE model by artificially introducing additive white Gaussian noise with varying signal-to-noise ratios (SNRs) into the dataset I. We compare DGIFE’s performance against representative benchmark models: EEGNet, DBConforme, Mixup, and URM. We systematically introduced additive white Gaussian noise at 4 distinct SNR levels into the test data: 20 dB (mild noise), 10 dB (moderate noise), 5 dB (severe noise), and 0 dB (extreme noise). As shown in Table 5, the experimental results clearly demonstrate that DGIFE exhibits superior noise resistance. As the SNR decreases, the performance of all models deteriorates. However, DGIFE maintains significantly higher ACC across all noise levels and exhibits a more gradual performance decline. This experiment directly proves that DGIFE’s feature purification and decoupling mechanisms effectively enhance its noise resistance capabilities.

**Table 5.** Experimental results of the model under different noise conditions, where the bold values indicate the best experimental results

Method	0 dB	5 dB	10 dB	20 dB	No noise
EEGNet	59.83 (↓17%)	60.14 (↓16%)	67.80 (↓6%)	68.10 (↓5%)	72.02
Mixup	61.61 (↓16%)	62.31 (↓15%)	63.13 (↓14%)	63.37 (↓14%)	73.28
DBConformer	**51.41 (↓10%)**	51.73 (↓9%)	**54.32 (↓5%)**	54.72 (↓4%)	56.90
URM	61.13 (↓13%)	68.96 (↓22%)	69.13 (**↓**19%)	**69.86 (↓0.9%)**	70.50
**DGIFE**	69.20 (↓11%)	**71.38 (↓8%)**	72.50 (**↓**6%)	74.03 (↓4%)	77.36

### Visualization and analysis of spatiotemporal features

To verify whether the DGIFE model can learn features consistent with neurophysiological principles and to gain deeper insight into its decision-making process, we analyzed the key spatiotemporal features identified by the temporal encoder and channel encoder modules. We visualized the multichannel convolutional weights of the temporal encoder module and the gated attention weights of the channel encoder module, aiming to reveal that the model can extract identical temporal and spatial features across different subjects.

#### Time-based visualization analysis

**Fig. 4.**
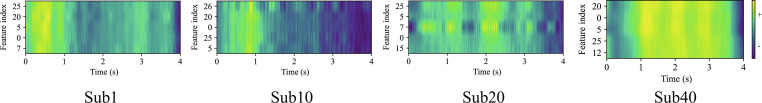
Visualization of time convolutional weights.

For the MI task, we visualized the temporal encoding weights across different subjects in dataset I using heatmaps. Fig. 4 displays the top 5 weighted EEG channels and their temporal variation patterns. Results reveal highly consistent distributions of high-weight channels across subjects, with weight values significantly increasing after task initiation and gradually decreasing toward task completion. This phenomenon indicates that the temporal coding module effectively captures shared temporal features across subjects and automatically aligns to task onset, thereby autonomously focusing on task-relevant information-dense periods.

#### Channel feature visualization analysis

**Fig. 5.**
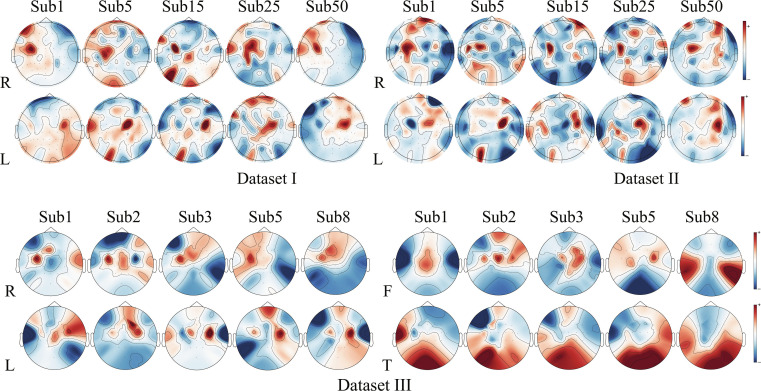
Visualization of channel weights. R, right hand motor imagery; L, left hand motor imagery; F, foot motor imagery; T, tongue motor imagery.

To validate the physiological plausibility of the channel coding module, we mapped its gated attention weights onto brain topographies, as shown in Fig. 5. Analyses are conducted on MI tasks using 5 subjects from each of 3 datasets. Results consistently showed that during right-hand imagery, the module’s attention significantly concentrated on the left sensorimotor cortex (electrode C3), whereas during left-hand imagery, attention focused on the corresponding right-brain regions (electrode C4). This distribution pattern fully aligns with the fundamental principle of contralateral control in the human brain and the neurophysiological phenomenon of ERD/ERS during MI. This indicates that the channel attention module effectively captures shared neural activity features across different subjects.

### Visualization

**Fig. 6.**
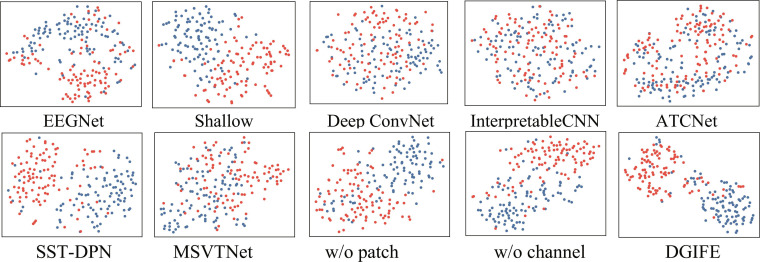
Scatter plots obtained with different models.

To verify the validity of our proposed model more visually, we use *t*-distributed stochastic neighbor embedding [39] to visualize and analyze the embedded features. The visualization results in Fig. 6 show the feature distributions of the comparison model and the ablation experimental model under the same subjects in dataset I before inputting the classification layer. The comparison results show that the DGIFE model has higher feature separation, further indicating that the model learns richer category features and significantly improves the classification performance.

## Conclusion

In this study, we proposed the DGIFE model designed to address the problem of domain bias that exists in a cross-subject task. The model consists of 2 main components: feature enhancement and feature decoupling. In the feature enhancement stage, we used a multigranularity feature extraction module and a channel self-attention module, both of which effectively improve the feature extraction capability of the model for diverse EEG data and are able to capture key features at different scales, thus enhancing the model’s expressiveness and generalization ability. In the feature decoupling part, we designed a generalized decoupling model, which constrains the feature extraction process by introducing multiple loss functions to ensure that the extracted features better reflect the cross-domain commonalities while avoiding overfitting phenomena. With this design, we can not only maintain high decoding ACC across tasks and datasets but also improve the cross-domain adaptability of the model, especially when there is a large offset in the data distribution. Our experimental results show that the DGIFE model achieves good performance on multiple datasets, especially when dealing with data with different subject and device variations, demonstrating its good generalization ability and stability.

## Data Availability

This study utilized 3 public databases. Giga and OpenBMI can be consulted on the website https://gigadb.org/, while BCIC-IV-2a can be accessed at https://www.bbci.de/competition/iv/#dataset2a.
